# Chronic Chagas' Disease: Targeting the Interleukin-2 Axis and Regulatory T Cells in a Condition for Which There Is No Treatment

**DOI:** 10.3389/fmicb.2016.00675

**Published:** 2016-05-13

**Authors:** Jose Mengel, Fabíola Cardillo, Lain Pontes-de-Carvalho

**Affiliations:** ^1^Laboratory of Clinical Immunology, Oswaldo Cruz Institute (FIOCRUZ)Rio de Janeiro, Brazil; ^2^Laboratory of Clinical Immunology, Faculty of Medicine of Petrópolis-FASERio de Janeiro, Brazil; ^3^Gonçalo Moniz Research Center, FIOCRUZSalvador, Brazil

**Keywords:** interleukin-2, CD25, regulatory T cells, *Trypanosoma cruz*, Chagas disease, IL-10

## Introduction

The intracellular protozoan parasite *Trypanosoma cruzi* causes Chagas' disease in humans (Mengel and Rossi, 1992; Rassi et al., 2010). According to the latest studies, about 5–8 million people are infected by this parasite around the world, representing a significant global economic burden (Rassi et al., 2010; Lee et al., 2013; Maguire, 2015). The infection may be divided in acute, indeterminate and chronic (Mengel and Rossi, 1992; Andrade et al., 2014). The acute disease, much less frequent nowadays, may be, in most patients, successfully treated with benznidazole, a drug that efficiently kills the parasite (Rassi et al., 2010). On the other hand, the chronic disease is by far the most important condition, concerning the total number of infected people. The chronic disease is characterized by a persistent inflammatory reaction and destruction of host cells, affecting mainly the peripheral autonomous nervous system in the gastrointestinal tract, the heart muscle and intracardiac nerves in ~30–40% of the infected patients, causing the development of megaesophagus and megacolon, and cardiomegaly associated with progressive and untreatable heart failure, in addition to an increased frequency of sudden death (Andrade et al., 2014). Yet, the majority (about 60–70%) of the patients that progress to the chronic phase remain clinically asymptomatic (Umezawa et al., 2001). Contrarily to most predictions, a large, multicentric, placebo-controlled, double-blinded clinical trial has shown that treatment with benznidazole has failed to modify the clinical outcome of chronic Chagas' disease, in spite of promoting a significant parasite load reduction (Morillo et al., 2015). these results argue that the treatment concept fails rather than the anti-parasite effect of the drug itself, suggesting that the presence of the parasite in the chronic infection may not be the only factor responsible for its clinical progression but perhaps a chronic infection that is inappropriately dealt with by the host defenses would be a major question, possibly involving a malfunction of regulatory immune mechanisms and autoimmune phenomena (Mengel and Rossi, 1992; Cardillo et al., 2015). This theoretical framework also accommodates most chronically *T. cruzi*-infected patients, where this malfunction of regulatory immune mechanisms would not take place, a balanced immune response would be achieved, and pathology would not prevail (Cardillo et al., 2015).

## Regulatory t cells in chagas' disease

Regulatory T cells may curb inflammatory responses, allowing a partially effective anti-parasite immune response to remain active. In fact, the role of CD4^+^CD25^+^Foxp3^+^ Treg cells during the chronic *T. cruzi* infection has been evaluated in mouse and humans. The most recent studies have suggested an important role for this T cell subpopulation in the chronic disease. For instance, recent findings in humans have shown an increased percentage of Treg cells in chagasic subjects in the indeterminate chronic phase of the infection (free of disease) when compared to patients with heart damage, suggesting an important role for Tregs in Chagas' disease (de Araujo et al., 2011).

In addition, *in vivo* Treg blockade by using nondepleting monoclonal antibodies to CD25, a marker for these cells, diminished the myocardial inflammation in the chronic infection, a finding that was partially ascribed to consequent increased levels of IL-2 secretion and expansion of CD4^+^CD25^+^ regulatory T cells (Nihei et al., 2014; Bonney and Engman, 2015), providing an indication that the functional activity of Treg cells might be of critical importance during the chronic phase of the infection. It should be noted that not all CD4^+^CD25^+^foxp3^+^ T cells in humans are suppressive and a fraction of them have other effector functions (Miyara et al., 2015). It was recently described that the expression of CD15s (sialyl Lewis x) identifies the most suppressive foxp3 high regulatory CD4^+^CD25^+^ T cells (Miyara et al., 2015), discriminating suppressive from effector CD4^+^CD25^+^foxp3^+^ T cells in humans. Interestingly, a previously published study has described that the expression of CD15s was decreased in peripheral blood lymphocytes from patients with severe Chagas' disease (Laucella et al., 2001). Therefore, the expression of CD15s in Tregs may be also relevant to predict progression to pathology or treatment efficacy in chagasic patients and further studies concerning this point are warranted.

Administration of nondepleting anti-CD25 mAb increases the production of IL-10 by T cells during the chronic *T. cruzi* infection in mice (Nihei et al., 2014). It is clear that IL-10 is a pro-survival cytokine during the acute infection, by controlling immune hyperactivation and/or by increase immunity to the parasite (Hunter et al., 1997; Roffe et al., 2012). In Chagas disease, IL-10 seems to be part of a regulated, nonpathogenic immune response in patients without cardiomyopathy (Poveda et al., 2014). These studies suggest that exogenous IL-10 supplementation may be helpful in the control of myocarditis during Chagas' disease. Pegylated IL-10 shows an increased *in vivo* half-life and helps in the activation of CD8 T cells. This activity would certainly be important in the control of residual tissue infection (Oft, 2014). Additionally, pegylated IL-10, through its potential antifibrotic activity (Mattos et al., 2012), would be helpful in the control of heart fibrosis, along *T. cruzi* infection, making exogenous IL-10 supplementation an extremely interesting approach to be tested in future studies.

## The interleukin 2 axis in immunity and autoimmunity

Interleukin-2 (IL-2) was first identified as a growth and differentiation factor produced and utilized by T cells (Liao et al., 2013). IL-2 is mainly produced by CD4 T cells and, upon activation, the production of IL-2 declines along with the T cell differentiation process (Sallusto et al., 2004). Therefore, memory T cells produce less IL-2 than naïve T cells. IL-2 is also produced to a lesser extent by CD8 and NK T cells, some dendritic and mast cells (Liao et al., 2013) There are two types of receptors for IL-2 (IL-2R), the high and the low affinity receptors (Liao et al., 2013). The low affinity receptor is a molecular complex formed by the association of the beta and the common gamma chains. The IL-2R beta chain (CD122) is expressed on T cells and many other cell lineages (Liao et al., 2013). The high affinity receptor has the addition of the IL-2R alpha chain (CD25) to the low affinity molecular complex (Liao et al., 2013). CD25 is mainly expressed in some activated T and B cell subpopulations as well as NK cells (Liao et al., 2013). IL-2 is of crucial importance for the T cell proliferation and survival during activation and differentiation to short-lived memory cells (Liao et al., 2013), being dispensable after the expression of high levels of the IL-7 receptor or the expression of the IL-15 alpha chain receptor (Liao et al., 2013). Therefore, it seems that IL-7 and/or IL-15 rather than IL-2 are the common gamma chain cytokines that help in the proliferation and maintenance of long-lived memory T cells (Xu et al., 2014). IL-2 is also a potent cytokine, helping CD8 T cell activation and differentiation to effector cells, participating in the process of NK cell activation and development of their lytic activity (Liao et al., 2013). On the other hand, IL-2 is involved in the regulatory arm of the immune response, by augmenting the proliferation of regulatory Foxp3^+^ T cells and the reinforcement of the FAS/FAS-L immunoregulatory pathway (Liao et al., 2013; Kosmaczewska, 2014). These two opposing functional activities seem to be related to the amount of the cytokine available in the microenvironment (Kosmaczewska, 2014). Therefore, low amounts of IL-2 would favor the immunoregulatory pathway, whereas high amounts would be required for driving effector immune responses (Kosmaczewska, 2014). The role of IL-2 in the immunoregulatory axis of the immune system is evident in mice and humans where the availability of IL-2 was reduced (Liao et al., 2013; Kosmaczewska, 2014). For instance, IL-2R-beta or IL-2R–alpha chain knockout mice develop autoimmune disease and blocking of IL-2 by *in vivo* treatment with monoclonal antibody to IL-2 accelerates autoimmunity in mice (Setoguchi et al., 2005). In humans, several polymorphisms in the IL-2 pathway have been linked to type I diabetes and loss of function of Foxp3^+^ Treg biological activities. In addition, low levels of circulating IL-2 have been associated with different autoimmune diseases (Yamanouchi et al., 2007). Chagasic patients with cardiomyopathy show decreased production of IL-2 by peripheral blood leucocytes (Briceno and Mosca, 1996). However, serum IL-2 levels have been reported to increase in chagasic patients with cardiomyopathy (Poveda et al., 2014). The reasons for the discrepancy are not clear, but IL-2 may be produced by cells trapped in other tissues and the presence of serum neutralizing factors, such as soluble serum CD25RA or anti-cytokine antibodies generated by a dysregulated immune response may reduce IL-2-bioactivity (Campen et al., 1988; Tsybikov et al., 2015). Soluble serum CD25RA and autoantibodies to many different self-antigens and structures have been described in Chagas disease (Moretti et al., 2002; Vicco et al., 2013). Autoimmune diseases usually proceed with lymphopenia and signs of T cell senescence (Stockinger et al., 2004; Thewissen et al., 2007). These characteristics may reflect a dysfunctional thymus and lower T cell output (Thewissen et al., 2005; Vieira et al., 2008). A similar picture has emerged for Chagas' disease. CD4 and CD8 T cells from chagasic patients carry markers of immunosenescence and present an exhausted functional phenotype with diminished production of IL-2 (Albareda et al., 2009, 2015). Increased levels of immunosenescence correlated with more severe forms of the disease (Cardillo et al., 1993; Albareda et al., 2009, 2015). Recent studies have also shown that the thymus is damaged during *T. cruzi* infection and therefore, lower numbers of Tregs and conventional thymic T cell emigrants would reach peripheral organs. This diminishes T cell renew along the infection similarly to what has been previously described in other classical autoimmune diseases (Gonzalez et al., 2016). Therefore, immunesenescence and autoimmunity would prevail in about 30% of infected people due to the low numbers and poor regulatory activity of Tregs that lack IL-2 produced by the pool of effector conventional T cells.

## Toward novel strategies to treat chronic chagas' disease

The study of acute and chronic phases of infection with intracellular pathogens, such as *T. cruzi*, allows the elucidation of the mechanisms and conditions that may be targeted to reprogram the host immune system by using tools that interfere with components of the regulatory arm of the immune system machinery. This knowledge would certainly result in a better understanding of the necessary balance to achieve or reestablish the health of the host during *T. cruzi* infection, thus providing new strategies to treat Chagas' disease. In this regard, the *in vivo* biological activity of nondepleting antibodies to CD25 seems to reinforce rather than inhibit the function of regulatory T cells either in mice or humans (Nihei et al., 2014; Huss et al., 2015). In addition, the *in vivo* administration of IL-2 or complexes of IL-2/anti-IL-2 to increase the numbers and functional activity of regulatory T cells would also be a valuable approach to be used in Chagas' disease (Letourneau et al., 2010). Low doses of IL-2 have already been used successfully in many clinical trials (Matsuoka et al., 2013; Kosmaczewska, 2014; Humrich et al., 2015). However, a consensus for the low-dose regimen of IL-2 administration has not been established and serious concerns about which dose of IL-2 to use in different clinical conditions remains open to debate (Perol et al., 2014).

The notion concerning the manipulation of Treg cells either by antibodies to CD25, IL-2, and/or IL-10 might open up a new avenue for therapeutic strategies in Chagas' disease. It should be noted that more pre-clinical studies, using different strains of *T. cruzi* in combination with distinct strains of mice should be performed in order to better establish these conceptual clinical interventions, perhaps in addition to more acquiescent schemes of benznidazole treatment (Bustamante et al., 2014).

## Author contributions

JM Design the paper hypothesis, wrote the first draft. FC Design the paper hypothesis, wrote the first draft. LP Design the paper hypothesis, wrote the first draft.

### Conflict of interest statement

The authors declare that the research was conducted in the absence of any commercial or financial relationships that could be construed as a potential conflict of interest.
